# The complete genome of *Xanthomonas citri* pv. *mangiferaeindicae* strain B3, isolated from diseased mango leaves in Guangxi, China

**DOI:** 10.1128/mra.00299-25

**Published:** 2025-06-16

**Authors:** Qiufei Ouyang, Cuifeng Yang, Lingling Lv, Jian Huang, Xi Li, Zhengjie Zhu

**Affiliations:** 1 Guangxi Key Laboratory of Biology for Mango, Baise University 372245, Baise, Guangxi, China; 2 College of Agriculture and Food Engineering, Baise University 372245, Baise, Guangxi, China; The University of Arizona, Tucson, Arizona, USA

**Keywords:** *Xanthomonas citri *pv. *mangiferaeindicae*, genome, pathogen, mango

## Abstract

In this study, we sequenced the complete genome of *Xanthomonas citri* pv. *mangiferaeindicae* strain B3, isolated from mango leaves with black spot symptoms. The 5.29 Mb genome comprises one circular chromosome and two plasmids, with a GC content of 64.5%, shedding light on its virulence and antibiotic resistance mechanisms.

## ANNOUNCEMENT

Mango bacterial black spot, caused by *Xanthomonas citri* pv. *mangiferaeindicae*, is one of the most destructive diseases threatening mango (*Mangifera indica*) production in Guangxi, China (1). In severely infected orchards, the disease causes severe yield losses, reduces fruit quality, and leads to significant economic damage (2).

To better understand the pathogen’s virulence mechanisms, resistance determinants, and potential control strategies, we sequenced the genome of strain B3, an isolate obtained from diseased mango leaves exhibiting typical bacterial black spot. Mango leaves showing characteristic bacterial black spot were collected from orchards in Baise, Guangxi, China (23.93 N, 106.65 E). Tissues at the lesion margins were surface-sterilized and then soaked in sterile water. The resulting suspension was streaked onto KC medium (yeast extract 7 g/L, peptone 7 g/L, glucose 7 g/L, agar 18 g/L, cephalexin 40 mg/L, kasugamycin 20 mg/L, and propiconazole 20 mg/L, pH 7.2) (3). Plates were incubated at 28°C for 3–5 days. Single colonies displaying mucoid, round, off-white characteristics were picked and restreaked on nutrient agar medium for at least three times until pure cultures were obtained (4). Following isolation, the strain was subjected to a pathogenicity test using detached, healthy mango leaves (5). For molecular identification, a single colony was picked from nutrient agar and grown overnight in 50 mL of nutrient broth at 28°C with shaking before DNA extraction. Following nutrient broth culture, the strain was preserved at –80°C in a 1:1 mixture of culture and sterilized glycerol. The genomic DNA of strain B3 was extracted by kit-based method, and the nearly full-length 16S rRNA gene was amplified using universal bacterial primers 27F and 1492R (6). BLAST analysis of the 16S rRNA gene sequence revealed 99.93% identity to *Xanthomonas citri* pv. *mangiferaeindicae* (GenBank accession no. EF989732.1), confirming the taxonomic status of strain B3.

The genome DNA of strain B3 was extracted using QIAamp DNA Mini Kit (QIAGEN, Shanghai, China). The quality and quantity of genomic DNA (gDNA) were determined using Nanodrop and Qubit 4 Fluorometer. Hybrid bacterial sequencing was performed using Oxford Nanopore Technology (ONT) with custom analysis and annotation, as described below. For ONT sequencing, we followed the Native Barcoding Amplicons protocol (EXP-NBD104, EXP-NBD114, and SQK-LSK109). For Illumina sequencing, fragmented genomic DNA was size-selected to 350 bp, and libraries were prepared using the Nextera XT DNA Library Preparation Kit. ONT and Illumina sequencing (paired end 150 bp) were run on PromethION and NovaSeq 6000, respectively. The ONT data were base called with Albacore software (v 2.0.1) and quality-controlled using LongQC (v1.2.0c) (7), resulting in a total yield of 1.41 Gb from 206,230 reads, with a mean read length of 6.84 kb and an average GC content of 64.08% at 238× coverage. Illumina reads were processed with fastp (v0.23.2) using default parameters (8). After filtering, 12,993,832 reads (1.89 Gbp) were retained. Using Flye to integrate both ONT and Illumina data for polishing (9), the final assembly yielded four contigs with a GC content of 64.67%, including a 5,171,865 bp circular chromosome, two circular plasmids measuring 85,600 bp and 33,072 bp, and one non-circular contig of 3,107 bp, respectively. To estimate the completeness and consistency of the assembled genome, CheckM analysis (v1.2.3) was used with the Prokaryotic Genome Annotation Pipeline (PGAP) gene set with the Xanthomonas, resulting in 97.9% completeness and 0% contamination. The National Centre for Biotechnology Information (NCBI) PGAP was used to annotate the assembled genome, resulting in 4,600 genes, 4,218 protein coding genes, 35 non-coding genes, 6 rRNA genes, 52 tRNA genes, and 286 pseudogenes (Table 1).

**TABLE 1:** Genome features of *Xanthomonas citri* pv. *mangiferaeindicae* strain B3

Features	Value
Host plant	*Mangifera indica*
Chromosome size (bp)	5,171,865
Plasmid_1 size (bp)	85,600
Plasmid_2 size (bp)	33,072
GC content (%)	64.5
Completeness (%)	97.9
Gene	4,600
Protein coding gene	4,218
Non-coding	35
rRNA	6
tRNA	52
Pseudogene	286
BioSample ID	SAMN47206023
BioProject ID	PRJNA1231277
Genbank ID	CP186917-CP186920

## Data Availability

This Whole Genome Shotgun project has been deposited in DDBJ/ENA/GenBank under BioProject accession no. PRJNA1231277 and BioSample accession no. SAMN47206023. The genome genbank accession numbers are CP186917–CP186920. The raw sequencing reads are available in the Sequence Read Archive (SRA) under accession numbers SRR32584364 and SRR32584365.
